# Qualitative approaches to use of the RE-AIM framework: rationale and methods

**DOI:** 10.1186/s12913-018-2938-8

**Published:** 2018-03-13

**Authors:** Jodi Summers Holtrop, Borsika A. Rabin, Russell E. Glasgow

**Affiliations:** 1 ACCORDS Dissemination and Implementation Science Program, and Department of Family Medicine, University of Colorado Denver School of Medicine, 12631 E. 17th Avenue, Aurora, CO 80045 USA; 2 University of California San Diego, La Jolla, CA USA

**Keywords:** Assessment, Qualitative, Re-aim, Evaluation, Methods

## Abstract

**Background:**

There have been over 430 publications using the RE-AIM model for planning and evaluation of health programs and policies, as well as numerous applications of the model in grant proposals and national programs. Full use of the model includes use of qualitative methods to understand why and how results were obtained on different RE-AIM dimensions, however, recent reviews have revealed that qualitative methods have been used infrequently. Having quantitative and qualitative methods and results iteratively inform each other should enhance understanding and lessons learned.

**Methods:**

Because there have been few published examples of qualitative approaches and methods using RE-AIM for planning or assessment and no guidance on how qualitative approaches can inform these processes, we provide guidance on qualitative methods to address the RE-AIM model and its various dimensions. The intended audience is researchers interested in applying RE-AIM or similar implementation models, but the methods discussed should also be relevant to those in community or clinical settings.

**Results:**

We present directions for, examples of, and guidance on how qualitative methods can be used to address each of the five RE-AIM dimensions. Formative qualitative methods can be helpful in planning interventions and designing for dissemination. Summative qualitative methods are useful when used in an iterative, mixed methods approach for understanding how and why different patterns of results occur.

**Conclusions:**

In summary, qualitative and mixed methods approaches to RE-AIM help understand complex situations and results, why and how outcomes were obtained, and contextual factors not easily assessed using quantitative measures.

## Background

The RE-AIM model was developed in 1999 in response to a need to have a framework to evaluate potential for, or actual, public health and population impact. [1] RE-AIM includes five dimensions to call attention to the importance of measuring not only a traditional clinical outcome (i.e. effectiveness), but also implementation outcomes that are less frequently assessed, but critical to producing broad impact. The RE-AIM dimensions (reach, effectiveness, adoption, implementation and maintenance) are outlined in Table 1. Since its development, there has been significant uptake in the use of RE-AIM as a planning and evaluation framework. [2] It has been used in over 430 publications [3, 4] that include both funded research and local and national programs. [5–7] There have been many more uses in proposed studies, although tracking is difficult. A recent review found that between 2000 and 2016, RE-AIM was the implementation science model used most frequently in grant applications to the NIH and to CDC. [8]

**Table 1:** RE-AIM Dimensions and Related Questions for Clinicians and Community Leaders

RE-AIM Dimension	Addresses
Reach	WHO is (was) intended to benefit and who actually participates or is exposed to the intervention?
Effectiveness	WHAT is (was) the most important benefits you are trying to achieve and what is (was) the likelihood of negative outcomes?
Adoption	WHERE is (was) the program or policy applied and WHO applied it?
Implementation	HOW consistently is (was) the program or policy delivered, HOW will (was) it be adapted, HOW much will (did) it cost, and WHY will (did) the results come about?
Maintenance	WHEN will (was) the initiative become operational; how long will (was) it be sustained (Setting level); and how long are the results sustained (Individual level)?

Note: Terms in parentheses are phrased for post intervention evaluation. The basic questions are phrased for use in program or policy planning. Adapted from Glasgow and Estabrooks (2017). [13]

RE-AIM has been used for both planning [2] and evaluation. [3, 9] Although the model is somewhat intuitive, full use of it requires in depth information and understanding of multi-level and contextual factors. [10] RE-AIM is one way to approach the “ultimate use” question of what intervention (programs or policies) components, conducted under what conditions and in what settings, conducted by which agents for which populations (and subgroups) are most effective in producing which outcomes; and for what cost, and under what circumstances? [11].

Although not always explicitly stated, full use of the RE-AIM model [10] necessitates use of both qualitative and quantitative methods to understand reasons for results on each dimension of RE-AIM. However, most studies rely heavily on quantitative methods and lack qualitative contributions. The published literature reveals a lack of qualitative methods use with RE-AIM. [3, 10] In the review of published empirical studies on RE-AIM by Gaglio and colleagues, among publications that reported on a given RE-AIM dimension, only 3.5–15.6% of the articles (median of 7%) included a qualitative measure. [3] A separate, more recent review by Harden and colleagues of behavioral intervention studies using RE-AIM revealed use of qualitative methods in 6–24% of studies across dimensions (median of 15%). [12]

Qualitative measures are of value in RE-AIM (and other planning and evaluation approaches) for several reasons. Some questions simply cannot be answered with quantitative data. Pulling data from an electronic medical record (EMR), analyzing a survey scale or counting does not work for some questions, or are too expensive to collect feasibly. Second, qualitative data provide answers to not just what happened, but why and how. They can illuminate patterns of results and why and how results were obtained for various outcomes, including unintended effects. Third, they provide diverse and multiple assessment methods to provide convergent validity for quantitative results. They can engage the participants in a collaborative manner and consider their inputs to a program or policy in a way that quantitative approaches do not. Finally, as with many research questions and evaluation approaches, having quantitative and qualitative methods for RE-AIM dimensions allows these methods to iteratively inform each other. This should enhance understanding and lessons learned, ultimately leading to better dissemination of evidence-based approaches into practice.

The purpose of this article is to summarize and recommend qualitative approaches to address the RE-AIM model and its various dimensions. We provide guidance for researchers and community groups that wish to use qualitative methods in their RE-AIM applications.

## Methods

### Applying Qualitative Methods to RE-AIM Dimensions.

Qualitative research provides meaning and understanding. It is utilized in both exploratory and explanatory research. This is in contrast to quantitative methods that utilize numbers and address statistical outcomes. In general, qualitative methods help understand how and why results on various individual RE-AIM dimensions, or patterns of results across dimensions (e.g. high reach and low effectiveness) occur. A wide variety of qualitative techniques and approaches can be used to address RE-AIM issues. As the focus of this paper is not to provide a comprehensive description of qualitative data collection and analysis methods, we refer the reader to excellent texts. [13–16] Instead of using one strategy, the methods selected should be tailored to the setting, research questions and resources available. Table 1 provides simple translational questions that can be used to inquire about RE-AIM issues by and with clinicians and community members. [17] In summary, there are a variety of methods conducive to qualitative work in exploration of RE-AIM dimensions. These include interviews, observations, focus groups, photovoice, digital storytelling, and ethnography. Analysis methods are also varied and dependent on the research or evaluation issue and question. Choices include grounded theory, thematic analysis, matrix, and immersion crystallization. Below we describe how qualitative methods can be used to address each RE-AIM dimension and key issues involved. Table 2 provides examples of questions and possible qualitative methods for each RE-AIM dimension.

**Table 2:** RE-AIM Elements and Qualitative Data Questions and Examples

RE-AIM Element	Questions/Description	Example
Reach	What factors contribute to the participation/non-participation of the participants? What might have been done to get more of the target audience to participate?	Focus groups and/or interviews at baseline and post-program to determine contributors to use of the program. Consider user-centered design and acceptability principles.
Effectiveness	Did the intervention work to effect the outcomes noted? What other factors contributed to the results? Are the outcomes found accurate? Are the results meaningful?	Ethnography in the setting to observe and reflect on outcomes as they are occurring. Key informant interviews to add participant reflections on the observed outcomes.
Adoption	What factors contributed to the organization and its individuals taking up the intervention? What barriers interacted with the intervention to prevent adoption? Was there partial or complete adoption? Why did some staff members in these organizations participate and others did not?	Key informant interviews with organizational leaders and “on the ground” implementers to identify their intentions and concerns before the intervention; during it, to understand adoption barriers and facilitators in real time; at the end to explain level of adoption and reflect on the intervention experience. To glean multiple perspectives on adoption from those who did and did not adopt and to what extent.
Implementation	How was the intervention implemented? By whom and when? What influenced implementation or lack of implementation? What combination of implementation effects affected the outcome results? How and why was the program or policy adapted or modified over time?	Photovoice with participants as they move through the intervention and their experiences. Critical incident analysis during the intervention to determine interaction of the intervention with contextual and personal factors. Observation at baseline and during the intervention as a fidelity check and to deepen understanding of issues as they emerge in practice.
Maintenance	Is the intervention being implemented (and adapted) after the intervention core period? What is sustained, what discontinued, what modified- and why?	Interviews and observation post-program to determine if the intervention is continuing and why.

#### Reach.

Standard means of assessing reach are to describe the number and percent of participants who participate in a desired initiative. From a qualitative method perspective, key issues concerning reach are understanding why people accept or decline participation and describing characteristics of participants versus non-participants that are not available from quantitative data or records. For example, if the desired goal is to reach all patients with diabetes and a hemoglobin A1c level over 8, the quantitative measure of reach would be the number or percent participating in the initiative out of the total eligible. Knowing that 25% of patients are participating provides insight into what degree of penetration occurred as a result of the initiative, but does not help to understand situations in and characteristics of the reached population that distinguish them from non-participants. Often, quantitative approaches have been used to describe reach in terms of the demographics of the reached versus non-reached population. For example, maybe the reach was 25%, but three quarters of the participants were female, Caucasian, and privately insured. Thus, the reach for this program largely misses Medicaid insured participants of both genders. These data represent identifiable characteristics of participants that provide a more comprehensive picture of who is missing. However, there are often characteristics that impact participation versus non-participation that are not routinely collected, readily available from EMRs or other databases, or not easy to quantify. Perhaps reach is limited by factors such as lack of trust in health care providers, disinterest in medication taking, or social determinants barriers faced by non-participants such as lack of transportation or family support to participate. These factors are difficult to ascertain without qualitative inquiry. To thoroughly understand reach, it is often necessary to conduct more in-depth and qualitative work to identify root cause issues of suboptimal reach.

#### Effectiveness.

Effectiveness focuses on important clinical or behavioral outcomes of interest and is most frequently summarized quantitatively. Key qualitative issues relevant to effectiveness are understanding whether various stakeholders find the effectiveness findings meaningful, why interventions produce different pattern of results across different RE-AIM dimensions, reasons for differences in results across subgroups, and why unanticipated negative results are observed. Continuing the example above with effectiveness being the objective of lowering hemoglobin A1c for patients with diabetes; assume that 50% were able lower their A1c to under 8, and that the mean A1c reduction from the intervention was 0.8%. However, this is just the beginning of an answer to the question: “was intervention X effective?”

Qualitative methods can contribute to assessing effectiveness in several ways. The first is understanding whether quantitative effectiveness findings are meaningful to various stakeholders (e.g., clinicians, patients). By meaningful we mean two things. First, if the measured outcome is valuable to the stakeholder (i.e., it provides them with information that helps them make decisions and/or achieve their respective goals). Second, if the actual quantitative change is meaningful enough to make the intervention worthwhile. In other words, what outcomes are of value to what stakeholders and how did the intervention fare on these factors? In our scenario, the question might be, was an average reduction of A1c by 0.8% meaningful for clinicians and participants?

Corollary qualitative questions include “Is reduction in A1c levels an appropriate indicator of an effective intervention for managing diabetes for the clinician and the patient? Does this provide them with information to decide what to do next and whether they are approaching or achieved their personal (i.e., patient) or practice (i.e., clinician) goals?” Second, is the amount of change (0.8% A1c reduction) sufficient effectiveness to make the intervention worthy for routine use? Does this change lead to sufficient improvement in the participants’ everyday life or quality of life to make participation in the intervention worthwhile? Although surveys can be utilized to answer some of these questions, they often lack the depth of the responses to be insightful.

Qualitative measures add understanding to differential or heterogeneous results. Quantitative data using subgroup analyses might identify subgroups of participants who were more successful in achieving A1c reductions. Qualitative methods are best suited to answer questions of why and how these groups are different in their level of success. Quantitative analyses alone are unlikely to identify more nuanced, sociocultural, or practical features that are major contributors to program effectiveness. In our example, we might find that those participants who perceived the intervention more favorably were able to experience better results than those who had more initial reservations toward the diabetes initiative.

With any intervention, in addition to planned, intended effects, there may also be unintended effects or consequences, either positive or negative. These are very relevant results and it is important to understand the total pattern of results, both intended and unintended. Qualitative methods can help identify some of the unintended outcomes which might not have been measured quantitatively, but can emerge as qualitative reports from both clinicians and participants, or identified through observations. In our example, observations conducted by the research team during implementation might reveal that as a result of participating in the diabetes intervention, patients get to spend less time with their physicians during the visit to discuss other medical concerns and/or physicians are less likely to initiate discussion about the emotional/mental health status of the patient (i.e., shift in priorities during visit).

#### Adoption.

Adoption is quantitatively operationalized as the number or proportion of settings and implementing staff who agree to participate in the intervention. The qualitative key issues in adoption parallel those of reach, but are at levels of settings and staff/implementers. It is important to understand why different organizations - and staff members within these organizations - choose to participate or not; and to understand complex or subtle differences in those organizations and staff members in terms of underlying dynamics and processes. For example, compatibility with mission and current priorities, external factors, and changing context (e.g., policy changes, new regulations, competing demands) often impact why organizations and key agents within an organization choose to participate or not. Quantitative methods can be used to identify standard organizational characteristics associated with participation (e.g., size, prior experience with related innovations, employee turn-over rates), but cannot provide a full or detailed understanding of key and usually unmeasured issues. Often empirical data are not available on key organizational factors (e.g., leadership, reasons for trying a new program).

Qualitative methods are extremely instructive for understanding reasons for adoption or lack of adoption across targeted staff and their settings. To identify a staff member’s rationale for participating or not in an initiative, semi-structured interviews can be extremely illuminating. Such questions can range from more superficial and straightforward interview questions such as “Please tell me about thoughts about participating in initiative X. Why did you not participate in initiative X?” to more in-depth probing with specified interview techniques that get deeply and in a detailed way, at specific factors related to uptake of the intervention.

For example, cognitive task analysis [18] is a collection of methods that allow much greater understanding of the organizational representatives in terms of their thinking about an issue, including how they make decisions as a group. Central is the concept of mental model, or how one conceptualizes what something is and how it will work. [19] Such issues are critical to understanding decision making around participation and commitment to participation. A key aspect of interviews for understanding adoption is to purposefully select key informants that speak from different perspectives. This includes individuals “in the trenches” with little authority to organization leaders and those fulfilling different tasks to provide triangulation among roles for a broader, deeper understanding.

Beyond interviewing, observation can often prove insightful in understanding forces underpinning adoption. Observation may include a tour of the physical site to see the layout, structure and space; it may include participant observation and/or role shadowing in which observation occurs with interaction with participants to explain what is happening and why. A formal ethnographic approach may or may not be used. Observation paired with interviews, if possible, is likely highly valuable because it may reveal inconsistencies between participants responses to interview questions and what they actually do in practice.

In our example of the diabetes intervention, perhaps the intervention was taken up by the three physicians and not the two physicians assistants. Interviews and observation could reveal that the physician assistants in this setting only provide care for patients in acute situations and thus do not have the opportunity for referral to a diabetes management program. Examining adoption on a qualitative level allows for greater understanding of the factors influencing adoption at both organizational and staff levels.

#### Implementation.

Implementation is quantitatively measured through indicators which include fidelity to the intervention protocol, adaptations made to the original intervention or implementation strategies, the cost (especially replication cost) [20, 21] to deliver the program, and the percent of key strategies that are delivered. There are many sub-issues in investigating implementation that lend themselves to qualitative inquiry. In fact, implementation is the dimension of RE-AIM where the need for qualitative understanding is most needed and often more meaningful than that of quantitative information. These issues include understanding the conditions under which consistency and inconsistency are occurring across staff, setting, time, and different components of program or policy delivery.

The traditional view of understanding implementation is that of fidelity. [22, 23] Knowing the extent to which fidelity (e.g. delivery of key components of a program) is achieved is an important aspect of understanding the contribution of the intervention to observed outcomes. If an effective intervention is not implemented well, then it is likely that its effects are diminished. Fidelity is usually measured by having delivery staff or observers complete checklists noting which intervention core components are delivered. While useful, additional inquiry utilizing qualitative methods is often necessary to understand the how, why and to what extent questions regarding implementation of an intervention.

Implementation can be understood at a deeper level using specific interview techniques to have a participant walk through step by step with an actual recent patient and then answer questions in multiple passes about the people involved, communication involved, tools and resources needed, and other aspects. Implementation may also be understood better through observation and shadowing with extensive field notes to observe what people do as they go through their day. In our diabetes example, perhaps a research assistant shadows the diabetes educator and discovers that many patients are only getting two sessions instead of the recommended four sessions. Interviewing reveals that the diabetes educator is overwhelmed with too many patients and has managed by reducing the number of contacts with patients.

In understanding nuances in implementation, [23] the importance of documenting and understanding adaptations is becoming increasingly recognized. [24, 25] In studies of scale-up or replication, interventions are almost never delivered and integrated precisely the way they were in prior efficacy studies or intervention guides. Increased understanding is needed of how and why programs are altered over time, by whom, for what reasons, and with what results. Qualitative methods, together with quantitative data in an iterative, mixed methods approach can be essential to understanding adaptations. [26] Adaptations are often not negative, and in fact, in many cases making adaptations to improve the fit between the intervention and local context improve the outcomes of the intervention in their own setting. [27, 28] Understanding not just what was adapted, but when, why and by whom will provide far greater information about the contributions to the outcomes as well as provide guidance for future scale up efforts. Finally, understanding decision maker perspectives on types of costs, resources and burden involved in delivering a program, the person or organization’s values, and how they construe “return on investment” is important when implementing, adapting or discontinuing (de-implementing) programs and can often best be illuminated through qualitative methods. [29]

Implementation issues are ideal for qualitative methods. If resources permit, an iterative combination of written survey responses to standard questions about the intervention and its implementation along with interviews of key informants and observations (such as shadowing roles) can be triangulated to inform a very thorough picture of not just how well an intervention is implemented, but why and how. This information is extremely useful in informing how the intervention may be translated to other settings and how barriers and difficulties can be overcome.

#### Maintenance.

Understanding program sustainability and the reasons why a) individual benefits continue or fade, and b) why the organization delivering the intervention decides to continue or discontinue the intervention are important for future program design and scale up. Maintenance is often not assessed as grant funding runs out and sustainability suffers. [3] Therefore, planning for sustainability beyond grant funding is an important issue to address both in the initial intervention design and implementation strategies planned, as well as after the formal evaluation of a new intervention is over. This is increasingly required and especially important in pragmatic studies. [30] Qualitative methods, coupled with early and ongoing stakeholder engagement throughout a study, can help illuminate sustainability problems early and allow implementers to plan for it and address it as needed. Also, brief interviews with those who continue or discontinue, or adapt an intervention can be very informative. In our example of an intervention for diabetes, we might use interviews with stakeholders to identify existing infrastructure that could support the ongoing use of the intervention and embed the intervention into this infrastructure. Case studies could describe how successfully sustained interventions in their context may provide lessons learned for others on how planning for sustainability can be done.

## Complex Issues and Challenges Involving the Use of Qualitative Approaches with RE-AIM

Above we have summarized basic application of qualitative methods to the various RE-AIM dimensions. Below we discuss more complex issues and challenges involving the use of qualitative approaches with RE-AIM.

### Understanding patterns of results across RE-AIM Dimensions.

One of the most challenging issues in applying RE-AIM, or other evaluation models, is understanding patterns of results. Programs or policies often produce different patterns of results across RE-AIM dimensions. For example, a program may be high in reach, but low in effectiveness; or be widely adopted, but poorly implemented. Qualitative methods can help “look under the hood” and understand relations among and reasons for differential outcomes on RE-AIM dimensions across programs or organizations. This then can be used to address low performing dimensions through different or modified implementation strategies. For example, we might see that an intervention has high reach and adoption rates, but it scores low on the implementation dimension. The reason behind this variation can be multifold including that the intervention might resonate with providers and participants initially, however, as implementation starts, it proves to be more complex than anticipated; or organizational support/resources might be lacking which hinders full implementation.

### Assuring completeness of inquiry and understanding through use of relevant theories and frameworks.

It is often helpful to apply relevant theories or frameworks to guide qualitative assessment questions and to ensure that important issues are not left out. Theories provide a possible explanation for the order and magnitude of influence of factors that may effect change, whereas frameworks often provide the components for consideration, but not how they interact. [31] Many consider the differences between theories, models and frameworks a moot point, however, their importance can be found in how they may provide structure for examination of factors that may be useful in exploring implementation and sustainment. For example, the Consolidated Framework for Implementation Research (CFIR) [32] is a synthesis of theories or meta-framework that can guide exploration of the different buckets of issues. It provides “a menu of constructs that have been associated with effective implementation” [33] such as the inner and outer settings when considering contextual factors. Including qualitative methods such as observation and interviews, along with the associated analysis, will add insight into these various groupings of issues in a way that other methods do not. For example, understanding low reach into a particular population may have elements of not just understanding that population (characteristics of individuals), but also how the organization planned, promoted and implemented the intervention, along with the context (inner and outer setting). Such process or active ingredients theories can be used in combination with RE-AIM to more fully understand processes behind results on RE-AIM dimensions; or how RE-AIM measures are related to clinical outcomes, such as weight loss, and reductions in hemoglobin A1c, blood pressure or lipid levels. In particular, understanding context at different levels and through the lens of different theories and models [34, 35] can be very useful.

There are many relevant theories regarding what helps an intervention to be successfully adopted, implemented or sustained that can be useful in helping to frame qualitative assessments. For example, Normalization Process Theory [36] examines factors such as coherence (sense-making work), cognitive participation (relational work), collective action (operational work), and reflexive monitoring (appraisal work) that help an intervention become “normalized” or in other words, routine. [37] Examining the main domains of theories can guide an inquiry to make sure relevant factors for maintenance are not overlooked. For example, it may be that an intervention is not maintained because the leaders in the organization are not actively learning from their implementation and adapting to make needed improvements. [38] Normalization Process Theory could help make these deficits clear.

There are many theories and models about what components and conditions are necessary for successful implementation and dissemination. [39, 40] Some are general and apply to all areas such as the study of macrocognition, or how groups make decisions and work together in real-world environments. [19] There are context specific models such as the Chronic Care Model [41] that inform what it takes to implement chronic care interventions and approaches in health care settings. Often, organizational level theories such as Donabedian’s Quality of Care Model [42] or Bodenheimer’s Building Blocks of Primary Care [43] are useful in understanding adoption and maintenance. Models and theories can be used to generate questions and collect and analyze data in both quantitative and qualitative ways, but the richness of how the elements of these models or theories come together in each situation is often through qualitative methods.

### How and when to conduct qualitative inquiries.

Qualitative methods should be used whenever possible, at multiple points in the course of program or policy delivery. At the pre-implementation stage, it is useful for exploratory work in selecting or developing an appropriate intervention and implementation strategy(ies). It is helpful to consider each of the RE-AIM dimensions and how they may be impacted by a specified intervention. [2, 17] Qualitative methods are helpful for baseline assessment related to the RE-AIM dimensions to highlight differences or changes that may occur during delivery and maintenance.

It can be useful to use RE-AIM self-tests about estimated impact to follow-up on RE-AIM planning profiles and help with decision-making. [44] Qualitative assessments of RE-AIM issues can be especially useful in helping organizations design programs and policies. [45] Clinics or community leaders often must decide among alternative evidence-based programs or policies. The most widely used (and often oversimplified or poorly conducted) qualitative approach during program planning is an initial focus group to help inform a program design or implementation strategies. This is less expensive and time consuming than alternative approaches, but often fails to reveal root causes issues. It may also not truly engage patients or stakeholders as partners. We encourage readers to consider examining approaches such as photovoice, direct observations, individual interview with probes, user-centered design methods, or other approaches outlined in Table 2 as alternatives. An innovative approach to engaging stakeholders as true partners uses the framework of co-creation or co-production. [46]

Such assessments relate to the emerging focus on stakeholder engagement, which involve but should not be limited to patients and families. From a RE-AIM perspective, it is important to conduct assessments and identify key factors at multiple levels such as patients, families or community members, implementation staff (which may change over the course of an interventions), organization leaders, and external agents such as community leaders or health care plans. [47] With the Patient Centered Outcome Research Institute (http://www.Pcori.org) great interest and attention has been placed on engaging patients in research and making research methods and approaches patient-centered. Qualitative approaches can be ideal for patient and stakeholder engagement. They can be involved in forming the qualitative research questions, identifying appropriate interview or observation topics, and help with interpreting results. A plethora of literature is available in the area of community based participatory research which can guide these efforts. [48]

Qualitative inquiry during the intervention implementation is sometimes controversial. There is concern that asking questions and being involved during program delivery may cause a “reactive” intervention effect. Although an extensive discussion of this matter is beyond this paper, consideration of the data collection method and timing is warranted. Strategies such as sampling a subset of participants that is analyzed separately, such as with Critical Incident Analysis; [49] or non-invasive methods such as shadowing/observation are likely to produce an abundance of understanding at little risk of contamination. Additionally, innovative and less frequently used qualitative approaches can help with addressing challenges that arise during implementation. For example, rapid response and analysis techniques can be very useful relatively early on to guide adjustments needed or to inform intervention implementation in a positive way. One practical way is to incorporate qualitative data collection on a small scale and incorporate it into mini-learning opportunities such as through the use of quality improvement PDSA (plan, do, study, act) cycles (see http://www.ihi.org/resources/pages/tools/plandostudyactworksheet.aspx).

Finally there is qualitative assessment for explanatory purposes, which can occur during or after program implementation (i.e., maintenance phase of a study). The focus of this inquiry is to understand what came before and why. Decision making with regard to which or how many qualitative methods to use in a given project is dependent on many factors, several of which relate to the practicalities of purpose of the evaluation, state of the science, budget and time constraints, and expertise.

### Cost and time considerations with qualitative research.

As with other methods of research, collecting and analyzing qualitative data is not free from the burdens associated with the time and expense. We posit that qualitative research is a necessary component to conducting a thorough mixed methods evaluation that explores not only the results of a RE-AIM evaluation, but the why, when, and how. However, cost and time considerations may prevail. In this case, some qualitative information is better than no qualitative information. Focus groups may be more efficient for the research team than lengthy individual interviews. Sampling of roles rather than observing all roles may be necessary. Perhaps multiple methods (i.e., both individual interviews and observations) may not be possible. Thematic analysis may be less time-consuming than true grounded theory. More rapid qualitative approaches are being developed [50, 51] and if greatly curtailed qualitative methods are all that can be used, some (valid) qualitative measures are better than nothing, especially as is appropriate to the research or program evaluation questions at hand.

## Discussion

There is an important need for increased use of qualitative approaches with RE-AIM (and most other dissemination and implementation models). Indeed, there is an increasing appetite for studies that utilize both qualitative and mixed methods in health services delivery research. [50, 52–56] There is a lack of guidance in the literature on how to use qualitative approaches for dissemination and implementation. [52] Inclusion of qualitative approaches is necessary for ‘full use’ of the RE-AIM model. [3, 10] Qualitative approaches are often selected because there is not a good way to quantify issues, and because they also offer their own unique value. Most frequently the combination of both qualitative and quantitative methods can best address a certain issue. Qualitative methods can be of particular help in situations that are very complex or in which rigorous or unbiased quantitative data are not available or feasible. Even when strong quantitative data and analyses are available, qualitative methods enrich the understanding and conclusions via triangulation. Qualitative approaches may not represent the entire population, but add depth and meaning to facilitate understanding. Such methods can protect against a false assumption that a program or approach does not work, when it was truly an implementation failure [22, 57] as well as when, where and what types of adaptation are beneficial. [25, 26] Finally, qualitative methods can offer insight into what it will take to overcome implementation failures in the future.

Qualitative methods can both enhance and advance the usefulness of RE-AIM. Possibly the greatest contribution of qualitative methods to RE-AIM, and especially when based on theory, is their value in explaining why various RE-AIM results were obtained and how they came about. A potential limitation is that quality use of qualitative approaches can require expertise, resources and time that may not be possible in low resource or non-research community settings. This is also true of quantitative approaches, however. There are decisions to be made on the depth of inquiry and amount and type of analysis and the trade-offs with the time and money it takes to conduct and thoroughly report on qualitative results. In many non-academic applications, there is a continuum trading off precision with timeliness. Hopefully, program implementers are not dissuaded by the need to complete a particular length or type of analysis because even modest contributions are likely to be more valuable than none. Even in such cases, selected qualitative approaches can be part of pragmatic application of RE-AIM. [17, 58]

## Conclusion

In conclusion, there is an clear need for more examples of the use and reporting of qualitative approaches with RE-AIM applications [3, 10]. We hope that in the near future there will be a sufficient number of such uses in RE-AIM to conduct reviews and make recommendations on how to best use and integrate qualitative strategies, identify lessons learned, and create more specific and authoritative guidelines for their use.

## Abbreviations

CFIR: Consolidated framework for implementation research
DIS: Dissemination and Implementation Science
EMR: Electronic medical record
PDSA: Plan, do, study, act
RE-AIM: Reach, effectiveness, adoption, implementation and maintenance
